# Decreased Glycogen Content Might Contribute to Chronic Stress-Induced Atrophy of Hippocampal Astrocyte volume and Depression-like Behavior in Rats

**DOI:** 10.1038/srep43192

**Published:** 2017-02-24

**Authors:** Yunan Zhao, Qiang Zhang, Xiao Shao, Liufeng Ouyang, Xin Wang, Kexuan Zhu, Lin Chen

**Affiliations:** 1 Key Laboratory of Brain Research, Basic Medical College, Nanjing University of Traditional Chinese Medicine, Nanjing 210046, China

## Abstract

The involvement of brain glycogen in the progress of chronic stress-induced impairment of hippocampal astrocyte structural plasticity and depression-like behavior is yet to be clarified. The present study designed three experiments to determine the role of brain glycogen in the plasticity and behavioral consequences of chronic stress. Time course studies on brain glycogen, astrocytes, and behavioral responses to stress were conducted in Experiment 1. Chronic stress decreased the hippocampal glycogen levels, reduced astrocytic size and protrusion length in the hippocampus, and induced depression-like behavior. Glycogen synthase 1 mRNA in the hippocampus was silenced by lentiviral vector-based RNA interference (RNAi) in Experiment 2. This RNAi produced a lack of glycogen in the hippocampus, decreased the hippocampal astrocyte size, and induced depressive behavior in rats. The mechanisms of chronic stress-induced brain glycogen decrease were investigated in Experiment 3. Chronic stress promoted hippocampal glycogen breakdown and increased hippocampal glycogen synthesis. Results suggest that decreased glycogen content was associated with chronic stress-induced atrophy of hippocampal astrocyte size and depression-like behavior. Furthermore, the decrease of glycogen content in the hippocampus might be due to the compensation of glycogen synthesis for breakdown in an insufficient manner.

Astrocytes are star-shaped glial cells in the central nervous system (CNS). These cells play an important role in the normal functioning of the CNS. Astrocytes regulate brain homeostasis, support neuronal health and metabolism, and are actively involved in the signaling processes^1^. Recently, an increasing number of data demonstrate that astrocyte structural plasticity (such as astrocyte protrusion length, branching, and volume) is disrupted after long-term exposure to stress, which may be the underlying mechanism of stress-induced depression-like behavior^2^,^3^. In Tynan *et al*.’s study^4^, for example, three weeks of restraint stress produced profound atrophy in astrocyte process length, branching, and volume in the prefrontal cortex of rats. A recent study showed that traumatic stress was capable of altering astrocytic morphology in the hippocampus, as shown by the reductions observed in the total number of primary processes^5^. However, the mechanism underlying the impairment of stress-induced astrocyte plasticity is yet to be clarified because of the relative newness of this field of research.

Glycogen, a complex glucose polymer found in a variety of tissues, is normally considered to function as storage for glucose. Certainly, the function of glycogen in securing a constant blood glucose level under a variety of conditions in the liver is well recognized. Glycogen in the brain is located mainly in astrocytes in the hippocampus, striatum, and cortex^6^,^7^. Although brain glycogen content is limited to an average of 3 μmol/g to 12 μmol/g tissue, the role of brain glycogen has been associated with the preservation of neuronal function during energetically challenging states (hypoxia or hypoglycemia)^8^,^9^,^10^. Nevertheless, glycogenolysis also occurs during brain activation, indicating that brain glycogen has a role in supporting brain function in nonpathological conditions^11^,^12^.

In general, glycogen metabolism is profoundly affected by stress, and glycogen content in peripheral tissues is reduced during stress because of increased glycogenolysis and decreased glycogen synthesis^13^. An increasing number of data show that stress or glucocorticoids also decrease brain glycogen content. After sleep deprivation, glycogen levels decreased in the hippocampus of rats^14^. Surgical stress resulted in nearly a 30% reduction in brain glycogen^15^. Recently, Zhang *et al*. found that chronic corticosterone exposure reduced the hippocampal glycogen level^16^. Thus, we hypothesized that brain glycogen might be involved in the detrimental effect of chronic stress on astrocyte plasticity and depression-like behavior, as brain glycogen is affected by stress and plays a key role in astrocytic energetics.

Based on this hypothesis, three experiments were performed in male rats in the current study. First, the effects of stress on hippocampal glycogen, astroglial structural plasticity, and depression-like behavior were dynamically observed to obtain the proper duration of stress (Experiment 1). Second, we knocked down glycogen synthase 1 (Gys 1, which is widely expressed, unlike Gys 2, which is restricted to the liver) mRNA to produce the lack of glycogen in the hippocampus by lentiviral vector-based RNA interference (RNAi). We also observed whether the lack of glycogen might induce astrocytic morphology alteration and depression-like behavior (Experiment 2). Finally, the impact of chronic stress on hippocampal glycogen metabolism was investigated to clarify the mechanism of chronic stress-induced brain glycogen decrease by detecting mRNA and protein expression, protein phosphorylation, and activities of Gys 1 and glycogen phosphorylase (Gyp) (Experiment 3).

## Results

### Experiment 1: Time course of behavior, hippocampal glycogen, and astroglial structural plasticity response to chronic unpredictable stress

#### Three and five weeks of unpredictable stress reduced hippocampal glycogen level and induced depression-like behavior in rats

**Figure 1:**
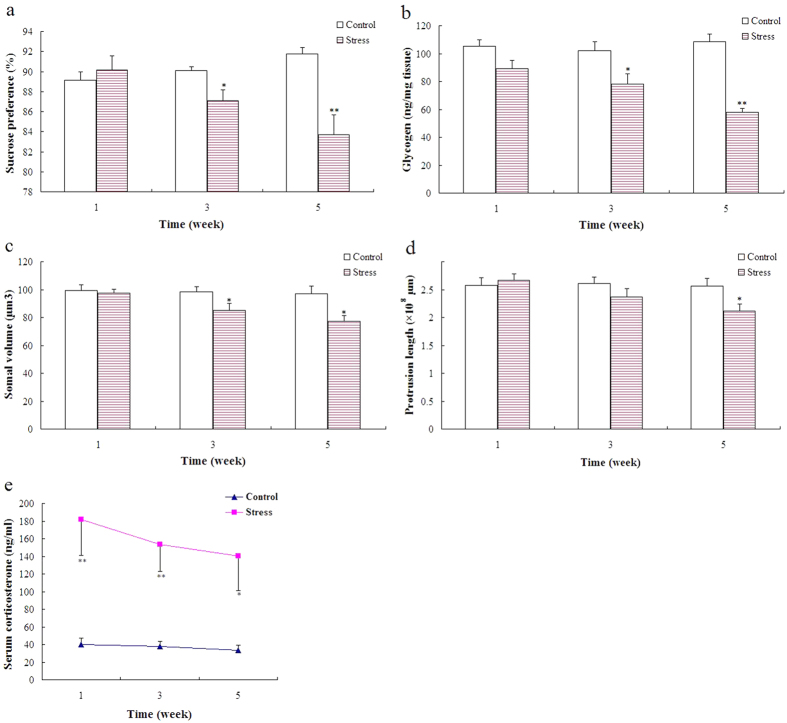
Time course of behavior, hippocampal glycogen, and astroglial structural plasticity response to chronic unpredictable stress. Depression-like behavior for each week was assessed in the sucrose preference (**a**) (mean ± S.E.M., *n* = 10). Hippocampal glycogen was assayed using a glycogen assay kit and presented here in μg glycogen per mg hippocampal tissue (**b**) (mean ± S.E.M., *n* = 5). Astroglial structural plasticity was assessed using somal volume (**c**) and protrusion length (**d**) of astrocytes (mean ± S.E.M., *n* = 5). Serum corticosterone was assayed using the AssayMax Corticosterone ELISA kit and presented here in the nanogram corticosterone per milliliter serum (**e**) (mean ± S.E.M., *n* = 10). A two-way ANOVA was followed by Student–Newman–Keuls *post hoc* analysis. **P < 0.01; *P < 0.05 for the stressed versus the control rats.

A significant effect of time (*F*_2,54_ = 9.104; *P* < 0.01) and treatment (*F*_1,54_ = 17.315; *P* < 0.01) was observed on sucrose preference, as well as a significant interaction between treatment and time (*F*_2,54_ = 2.898; *P* < 0.05). Multiple comparison tests further showed that one-week stress exhibited no obvious effect on sucrose intake, whereas three- (*P* < 0.05) and five-week (*P* < 0.01) stress significantly decreased sucrose preference (Fig. 1a).

The two-way analysis of variance (ANOVA) revealed significant changes in hippocampal glycogen levels, with a significant main effect of treatment (*F*_1,24_ = 18.272; *P* < 0.01) and time (*F*_2,24_ = 10.129; *P* < 0.01) and with a significant interaction between these factors (*F*_2,24_ = 5.821; *P* < 0.01). Multiple comparison tests further showed an obvious decrease in the hippocampal glycogen levels of rats after being stressed for three (*P* < 0.05) and five (*P* < 0.01) weeks (Fig. 1b).

#### Chronic unpredictable stress reduced hippocampal astrocyte structural plasticity in rats

The stereological estimates on the somal volume and protrusion length of glial fibrillary acidic protein (GFAP)-immunoreactive astrocytes are listed in Supplementary Table S1. A two-way ANOVA revealed a significant effect of stress treatment (*F*_1,24_ = 12.203; *P* < 0.01) and time (*F*_2,24_ = 3.415; *P* < 0.05) on hippocampal astrocyte size without a significant interaction between the two factors (*F*_2,24_ = 2.15; *P* = 0.120). Multiple comparison tests revealed that the somal volume of hippocampal astrocytes in the stressed rats decreased significantly at weeks three (*P* < 0.05) and five (*P* < 0.05) (Fig. 1c).

Regarding protrusion length of hippocampal astrocytes, no significant effect of time (*F*_2,24_ = 2.533; *P* = 0.099) and interaction was observed between treatment and time (*F*_2,24_ = 2.696; *P* = 0.088), but a significant effect of treatment (*F*_1,24_ = 4.850; *P* < 0.05) was observed. Multiple comparison tests further revealed that five-week (*P* < 0.05) stress significantly decreased the protrusion length of astrocytes. Additionally, three-week stress decreased the protrusion length of astrocytes (*P* = 0.081) (Fig. 1d).

#### Chronic unpredictable stress induced a sustained elevation of serum corticosterone levels

A two-way ANOVA revealed a significant effect of stress treatment (*F*_1,54_ = 33.187; *P* < 0.001) and time (*F*_2,54_ = 5.749; *P* < 0.01) on serum corticosterone levels, with a significant interaction between the two factors (*F*_2,54_ = 5.255; *P* < 0.01). Multiple comparison tests revealed that serum corticosterone levels increased significantly during weeks of stress (weeks 1 and 3, *P* < 0.01; week 5, *P* < 0.05) (Fig. 1e).

### Experiment 2: effects of Gys1 RNAi on astroglial structural plasticity and depression-like behavior

#### Silencing efficiency of the shRNAs against Gys 1 in the hippocampus of rats

**Figure 2:**
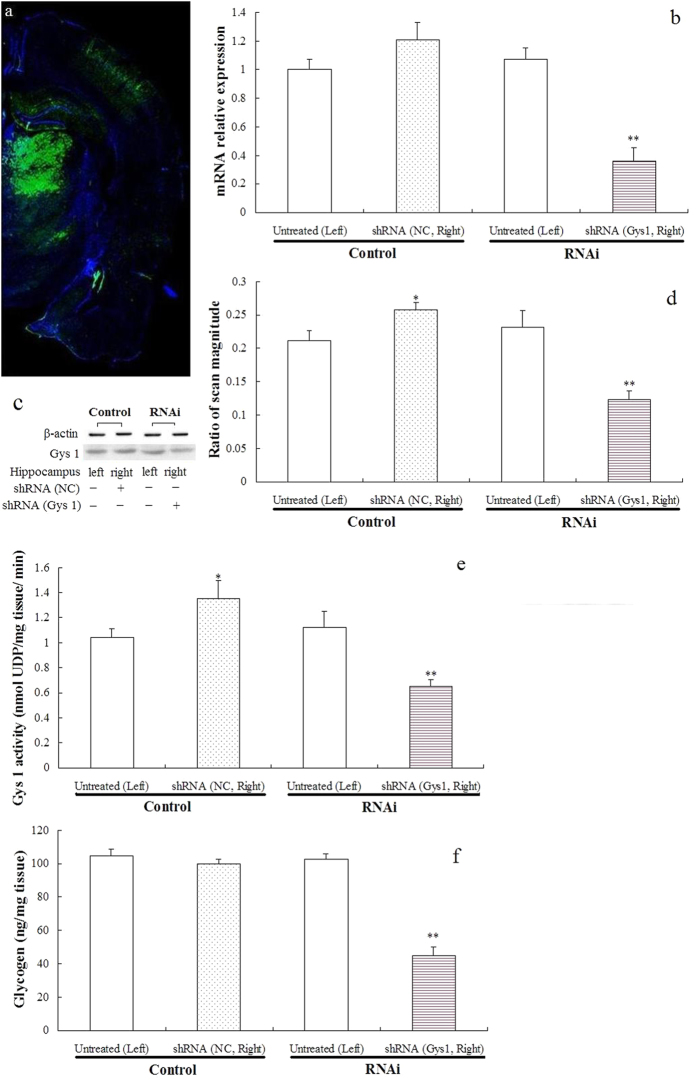
Silencing efficiency of the shRNAs against Gys 1. The right hippocampus of rats in the control and RNAi groups was injected with control lentivirus containing a NC sequence and recombinant lentivirus containing shRNA sequence targeting Gys 1. The lentivirus infection in the hippocampus was observed by EGFP positive expression (**a**). Hippocampal Gys 1 mRNA levels were determined by real-time PCR, and relative expression was calculated using the 2^−ΔΔCt^ method (**b**) (mean ± S.E.M., *n* = 5). Gys 1 protein levels were determined by Western blot analysis (**c**). The values of Gys 1 levels were normalized against the amount of β-actin (**d**) (mean ± S.E.M., *n* = 5). Gys 1 activity and tissue glycogen were measured and expressed as nmol UDP/mg hippocampal tissue/min (**e**) (mean ± S.E.M., *n* = 5) and μg glycogen per mg hippocampal tissue (**f**) (mean ± S.E.M., *n* = 5). Two-way ANOVA followed by Student–Newman–Keuls *post hoc* analysis. **P < 0.01; *P < 0.05, for the shRNA versus the untreated rats.

In general, the lentivirus infection was successful and was observed in the hippocampus, cerebral cortex passed by a needle, and brain regions under the hippocampus after two injections (Fig. 2a). The recombinant lentivirus decreased Gys 1 mRNA and protein expression, reduced Gys 1 activity, and decreased glycogen levels in the hippocampus (Fig. 2b–f).

A significant effect of infection (F_1,16_ = 5.569; *P* < 0.05) and RNAi (F_1,16_ = 4.667; *P* < 0.05) was observed on Gys 1 mRNA expression as well as a significant interaction between infection and RNAi (F_1,16_ = 4.825; *P* < 0.05). Multiple comparison tests further showed that the recombinant lentivirus significantly decreased the levels of Gys 1 mRNA by approximately 60% (*P* < 0.01) (Fig. 2b).

No significant effect of infection was observed on Gys 1 protein expression (*F*_1,16_ = 1.523; *P* = 0.396) and activity (*F*_1,16_ = 0.333; *P* = 0.794), but a significant effect of RNAi (*F*_1,16_ = 7.810; *P* < 0.05) (*F*_1,16_ = 10.133; *P* < 0.01) as well as a significant interaction between infection and RNAi (F_1,16_ = 12.825; *P* < 0.01) (*F*_1,16_ = 14.133; *P* < 0.01) were observed. Multiple comparison tests further showed that the control lentivirus significantly increased Gys 1 protein expression (*P* < 0.05) and activity (*P* < 0.05), whereas the recombinant lentivirus significantly decreased Gys 1 protein expression (*P* < 0.01) and activity (*P* < 0.01) (Fig. 2d,e).

A two-way ANOVA revealed significant changes in hippocampal glycogen levels, with a significant main effect of infection (F_1,16_ = 38.272; *P* < 0.001) and RNAi (F_1,16_ = 30.129; *P* < 0.001) and with a significant interaction between these factors (F_1,16_ = 31.821; *P* < 0.001). Multiple comparison tests further showed that the recombinant lentivirus significantly decreased the levels of hippocampal glycogen by approximately 50% (*P* < 0.01) (Fig. 2f).

#### Specific lentiviral shRNA-mediated knockdown of Gys 1 decreased hippocampal astrocyte size and induced depression-like behavior in rats

**Figure 3:**
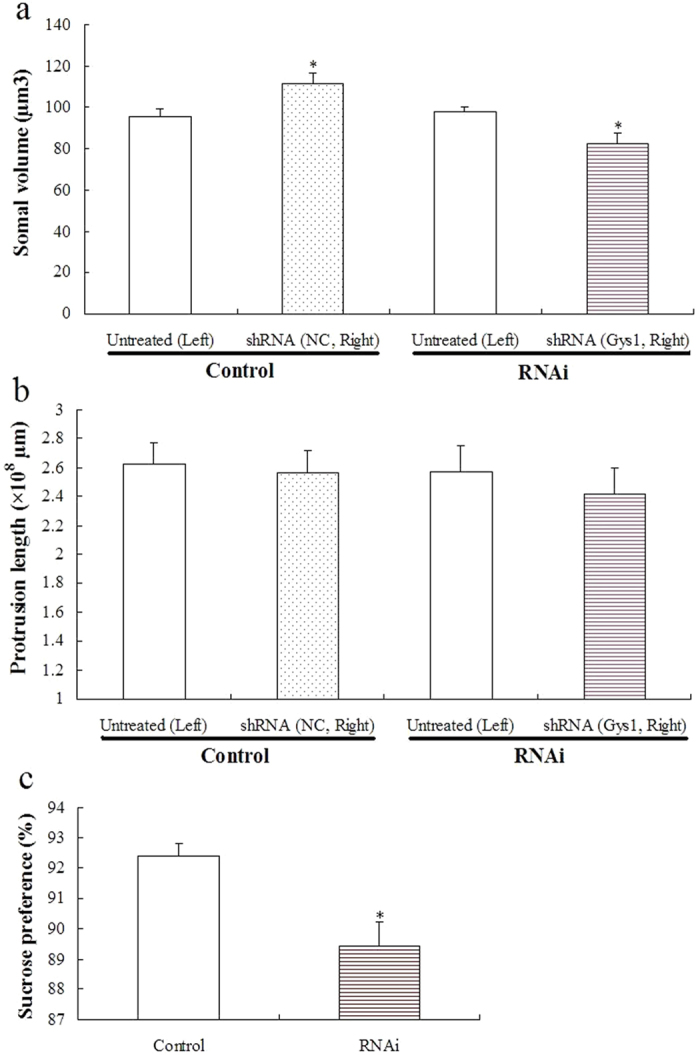
Effects of Gys 1 knockdown on somal volume (**a**) and protrusion length (**b**) of hippocampal astrocytes (mean ± S.E.M., *n* = 5) and sucrose preference (**c**) (mean ± S.E.M., *n* = 15). For somal volume and protrusion length, statistical significance was determined by a two-way ANOVA followed by Student–Newman–Keuls *post hoc* analysis. *P < 0.05 for the shRNA versus the untreated rats. A two-tailed Student’s *t*-test was used for sucrose preference. *P < 0.05 for the RNAi versus control.

The stereological estimates on the somal volume and protrusion length of hippocampal astrocytes are listed in Supplementary Table S2. A two-way ANOVA revealed no significant effect of infection (*F*_1,16_ = 0.005; *P* = 0.944) on astrocyte size, but a significant effect of RNAi (*F*_1,16_ = 10.340; *P* < 0.01) and a significant interaction between infection and RNAi (F_1,16_ = 14.344; *P* < 0.01) were identified. Multiple comparison tests further revealed that the control lentivirus significantly increased the somal volume (*P* < 0.05), whereas the recombinant lentivirus induced an obvious decrease in the somal volume (*P* < 0.01) (Fig. 3a). No obvious differences between groups were observed on the protrusion length of hippocampal astrocytes (Fig. 3b). Lentiviral shRNA-mediated knockdown of Gys 1 induced a significant decrease in sucrose intake in the sucrose preference test (*P* < 0.05) (Fig. 3c).

### Experiment 3: effects of chronic stress on hippocampal glycogen metabolism

#### Chronic stress promoted hippocampal glycogen breakdown

**Figure 4:**
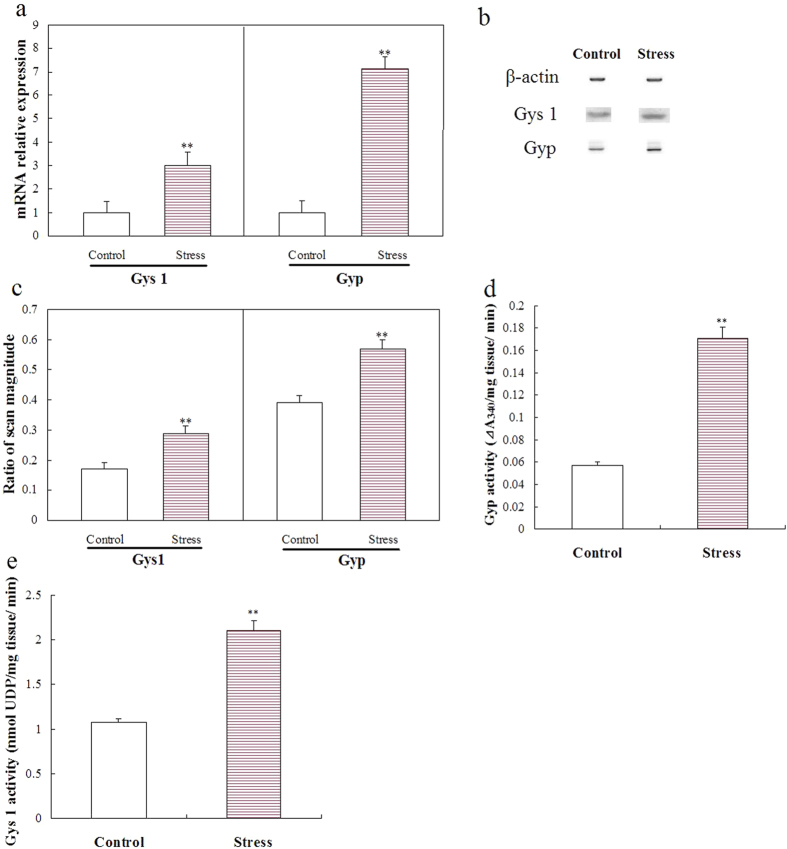
Effects of chronic unpredictable stress on Gyp and Gys 1 mRNA expression, protein levels, and activities. Gyp and Gys 1 mRNA levels were determined by real-time PCR. Relative expression was calculated using the 2^−ΔΔCt^ method (**a**) (mean ± S.E.M., *n* = 5). Protein levels were determined by Western blot analysis (**b**). The values of Gyp and Gys 1 levels were normalized against the amount of β-actin (**c**) (mean ± S.E.M., *n* = 5). Gyp and Gys 1 activity was measured and expressed as ΔA_340_/mg hippocampal tissue/min (**d**) (mean ± S.E.M., *n* = 5) and nmol UDP/mg hippocampal tissue/min (**e**) (mean ± S.E.M., *n* = 5). A two-tailed Student’s *t*-test was used. **P < 0.01 for the stressed versus control rats.

**Figure 5:**
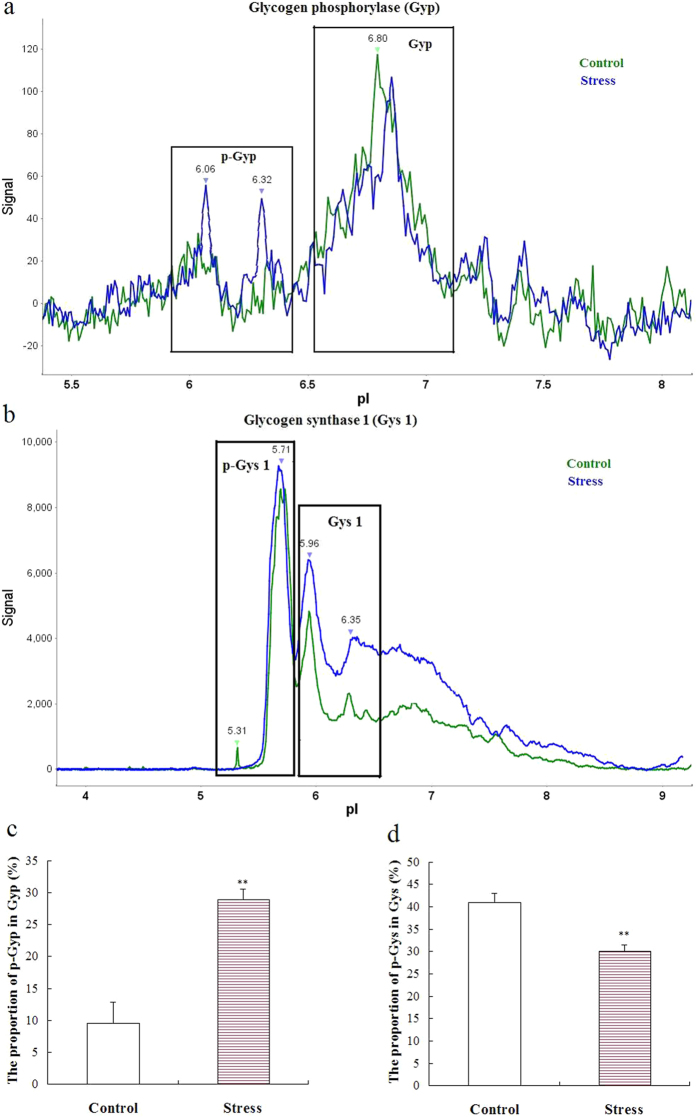
Effects of chronic unpredictable stress on Gyp and Gys 1 phosphorylation status. Protein phosphorylation was assayed by the NanoPro immunoassay method. The peaks at pI 6.02 and pI 6.32 disappeared after calf intestinal phosphatase treatment and were assigned to phosphorylated Gyp (p-Gyp) (**a**). Similarly, the peaks at pI 5.31 and pI 5.71 were assigned to phosphorylated Gys 1 (p-Gys 1) (**b**). Gyp and Gys 1 phosphorylation status was expressed as the proportion of p-Gyp in total Gyp (**c**) (mean ± S.E.M., *n* = 5) and p-Gys 1 in total Gys 1 (**d**) (mean ± S.E.M., *n* = 5). A two-tailed Student’s *t*-test was used. **P < 0.01 for the stressed versus control rats.

Hippocampal Gyp mRNA expression (Fig. 4a) (P < 0.01), protein levels (Fig. 4c) (P < 0.01), and activities (Fig. 4d) (P < 0.01) increased significantly after five weeks of stress. Hippocampal Gyp activities increased approximately threefold in the stressed group. The effects of stress on Gyp protein phosphorylation are shown in Fig. 5a. Chronic stress significantly increased Gyp phosphorylation levels (Fig. 5c) (P < 0.01).

#### Chronic stress increased hippocampal glycogen synthesis

Five-week stress induced a significant increase in hippocampal Gys 1 mRNA expression (Fig. 4a) (P < 0.01), protein levels (Fig. 4c) (P < 0.01), and activities (Fig. 4e) (P < 0.01). Hippocampal Gys 1 activities increased approximately twofold in the stressed group. The effects of stress on Gys 1 protein phosphorylation are shown in Fig. 5b. Chronic stress significantly reduced Gys 1 phosphorylation levels (Fig. 5d) (P < 0.01).

#### Chronic stress reduced the ATP levels and increased the AMP and ADP levels

Hippocampal ATP levels in the stressed group (2.142 ± 0.041 nmol/mg tissue) decreased significantly compared with the control group (2.381 ± 0.059 nmol/mg tissue) (*P* < 0.05). Meanwhile, five-week stress induced a significant increase of hippocampal AMP and ADP levels in the stressed group (0.091 ± 0.033 and 0.391 ± 0.063 nmol/mg tissue) compared with the control group (0.036 ± 0.024 and 0.262 ± 0.054 nmol/mg tissue) (*P* < 0.05).

## Discussion

### Astrocyte plasticity disruption and depression-like behavior induced by chronic stress

Chronic mild or traumatic stress is detrimental to the CNS. Stress is the major causal or exacerbating factor of depression in humans. Repeated stress can induce depression-like behavior in rodents^17^,^18^. In this study, we also found that three- or five-week stress reduced preference for the sucrose solution, which was frequently applied to evaluate depression-like behavior of rats induced by chronic stress. A wide body of evidence has shown that chronic stress alters the morphology of neurons, leading to changes in spine density and dendritic length^19^,^20^,^21^. These changes induced by stress have been used to explain the development of depression. However, astrocytes, which play a pivotal role in the normal functioning of the nervous system, are also an important target of stress.

Recent evidence has demonstrated that chronic stress also causes astrocyte morphological alterations, leading to atrophy of process length, branching, and volume in the prefrontal cortex or the hippocampus^4^,^5^,^22^. In this study, we also found that both three- and five-week stresses decreased the somal volume of astrocytes in the hippocampus of rats. Meanwhile, the protrusion length of astrocytes was influenced by stress other than the astrocyte volume. An obvious decrease was observed in the protrusion length of hippocampal astrocytes after being stressed for five weeks. Whether these astrocytic alterations play a role in the behavioral sequelae induced by chronic stress needs to be clarified. Antidepressant drugs that prevent stress-induced astrocyte changes were able to normalize stress-induced behavioral changes in Czéh *et al*.’s studies^22^. Additionally, astrocyte specific toxins induced similar behavior to those observed after stress exposure^23^,^24^, and astrocyte specific alterations in transgenic mice were able to emulate stress effects^25^,^26^. These findings suggest that astrocyte morphological disruption may underlie the behavioral consequences of chronic stress.

### Decreased hippocampal glycogen results in astrocyte volume atrophy and depression-like behavior

Glucose, glycogen, and lactate are traditionally identified with brain energetics, ATP turnover, and pathophysiology. However, recent studies showed that the metabolic trinity, glucose–glycogen–lactate, links astrocytes and neurons in brain energetics, signaling, memory, and gene expression^27^. In contrast to uptake and phosphorylation of glucose, the rapidity of brain glycogen breakdown contributes to the rapid energy requirements of brain activation. Glycogen mobilization can provide metabolic substrates to astrocytes *per se* as well as to neurons by the astrocyte–neuron lactate shuttle^28^. Recently, Duran *et al*. found that the lack of glycogen in the brain produced impairment in long-term memory formation and learning-dependent synaptic plasticity^29^. In this study, we found that hippocampal glycogen levels decreased after three and five weeks of unpredictable stress and were accompanied by impaired astrocyte structural plasticity and depression-like behavior. Whether decreased glycogen content is responsible for the plasticity and behavioral consequences of chronic stress must be verified further.

Consequently, we produced the lack of glycogen in the hippocampus of rats by Gys 1 mRNA knockdown to study the involvement of brain glycogen in astrocyte structural plasticity and depression-like behavior. We first determined whether lentiviral vector-based RNAi knocked down Gys 1 mRNA, resulting in the lack of glycogen. Our results showed that the lentivirus infection was successful in the hippocampus. The control lentivirus increased Gys 1 protein expression and activity, whereas the recombinant lentivirus decreased Gys 1 mRNA and protein expression. Hippocampal glycogen levels were decreased by approximately 50%. This value was close to that of the five-week stress that caused a reduction in glycogen levels. We then analyzed the functional consequences of the absence of hippocampal glycogen. The stereological analysis showed that the control lentivirus increased the somal volume, suggesting that the lentivirus infection itself might cause reactive astrocytosis. However, the lentiviral shRNA-mediated knockdown of Gys 1 obviously decreased the somal volume of hippocampal astrocytes by approximately 20%. The 50% decrease of the hippocampal glycogen might reduce the somal volume of astrocytes by more than 20% considering the infection-induced astrocyte reactivity. This condition indicates that brain glycogen plays a key role in the maintenance of astrocyte size. Additionally, we have attempted to increase glycogen to determine whether the overexpression of glycogen may salvage the stress-induced impairments; however, this procedure was not technically possible. Meanwhile, the lack of glycogen seemed to have no effect on protrusion length of hippocampal astrocytes within five weeks. This phenomenon suggested that other unknown mechanisms are involved in chronic stress-induced astrocyte protrusion length disruption.

Gys 1 knockdown induced depression-like behavior in rats, indicating that brain glycogen plays a key role in the maintenance of animal emotion. The presence of glycogen has been restricted to astrocytes; hence, our results may further clarify the contribution of astrocyte changes to the behavioral consequences of chronic stress. However, Saez *et al*. recently reported that neurons contained a low but measurable amount of glycogen and had an active glycogen metabolism^30^. Consequently, the effects of Gys 1 knockdown on animal emotion might also be due to changes in the neurons.

In addition, our study has one limitation that is inherent in the lentivirus infection. The hippocampus indeed showed an enhanced green fluorescent protein (EGFP) expression. However, other regions were also affected by the infection, particularly the thalamus, where the EGFP expression was most conspicuous. This condition could influence the behavioral results by affecting gustatory perception, as the thalamus is important for taste detection and recognition^31^,^32^.

### Different effects of chronic stress on peripheral and central glycogen metabolism

Repeated stress decreases glycogen content, especially in the liver and skeletal muscle, and induces hyperglycemia. Both our and other investigators’ studies showed that chronic stress or long-term corticosterone injection resulted in a reduction in brain glycogen in the CNS^14^,^15^,^16^. This condition indicates that repeated stress can induce a decrease in glycogen content, whether in peripheral or central tissues. However, the underlying mechanisms of glycogen content decrease seem to be dissimilar.

Stress stimulates the adrenal glands and pancreatic α cells to secrete epinephrine, norepinephrine, and glucagon. These hormones bind to adrenergic and glucagon receptors of peripheral tissues to produce cAMP, inositol-1,4,5-triphosphate, diacylglycerol and Ca^2+^. These second messengers activate a range of kinases to phosphorylate Gyp and Gys. Gyp is converted from the less active Gyp b conformation into active Gyp a conformation after phosphorylation to promote glycogenolysis. On the contrary, the phosphorylation on Gys inactivates it to inhibit glycogenesis^11^,^13^,^33^. Additionally, glucocorticoids as stress hormones inhibit insulin-stimulated glycogen synthesis^34^. Thus, the mechanisms of the decrease in chronic stress-induced glycogen content in peripheral tissues can be summarized as increased glycogenolysis and decreased glycogenesis.

To our knowledge, little research has been conducted on the effects of chronic stress on the glycogen mechanism of the brain. In this study, we found that five weeks of unpredictable stress increased Gyp mRNA expression, protein levels, and activity in the hippocampus. Meanwhile, we used the NanoPro immunoassay method^35^,^36^, which combines isoelectric protein focusing and anti-Gyp antibody detection, to measure accurately Gyp phosphorylation levels under the circumstance of limited anti-phosphorylated Gyp antibodies. We found that the total phosphorylation levels of Gyp were increased after five weeks of unpredictable stress. Additionally, we found that five weeks of unpredictable stress reduced ATP levels and increased AMP levels in the hippocampus. This result indicates energy deprivation because of the stress-induced increase of brain energy demand. Gyp is highly allosterically activated by AMP. Moreover, the regulation of Gyp reflects the role of glycogen breakdown in providing fuel for the cell during local energy deprivation. These findings suggested that chronic stress also increased glycogenolysis in the CNS. This result was similar to the effects of chronic stress on glycogenolysis in peripheral tissues.

However, the effects of repeated stress on hippocampal Gys were different from those of repeated stress on Gys in peripheral tissues. In this study, we found that five-week stress induced an increase in hippocampal Gys 1 mRNA expression, protein levels, and activity. The NanoPro immunoassay showed that the total phosphorylation levels of Gys1 decreased after five weeks of unpredictable stress. These data indicated that astrocytes in the hippocampus increase glycogen synthesis while suffering from repeated stress instead of inhibiting glycogenesis. Thus, chronic stress-induced decrease of glycogen content in the hippocampus might be due to compensation of glycogen synthesis for breakdown in an insufficient manner. The reasons underlying the different effects of stress on Gys in the peripheral and central tissues are worthy of further study.

## Conclusion

Overall, we have shown that chronic stress causes glycogen content decrease in the hippocampus because of insufficient compensation of glycogen synthesis for depletion. This condition is responsible for chronic stress-induced atrophy of astrocyte volume and depression-like behavior in rats. To our knowledge, this is the first study wherein the contribution of brain glycogen to astrocyte structural plasticity and animal depressive behavior has been suggested by means of RNAi tools. These findings may have relevant implications in treating stress-related emotional disorders.

## Materials and Methods

### Experimental animals

Male Sprague–Dawley rats (Laboratory Animal Center of Nanjing Medical University, Nanjing, China) weighing 200 g to 210 g at the beginning of the experiments were housed in groups of five in home cages made of Plexiglas material (45 cm × 30 cm × 15 cm) with sawdust on the floor and with food and water freely available, except as described below. Rats were maintained under a standard dark–light cycle (lights on between 7:00 and 19:00 h) at a room temperature of 22 ± 2 °C. Rats were adapted to daily handling during the week after delivery prior to the experiments. Experimental animals were weighed and randomly assigned to experimental groups. Experiments were performed during the light period of the cycle. All animal procedures were approved by the IACUC (Institutional Animal Care and Use Committee) of Nanjing University of Traditional Chinese Medicine and carried out in accordance with the Guidelines of Accommodation and Care for Animals formulated by the Chinese Convention for the protection of vertebrate animals used for experimental and other scientific purposes. All efforts were made to minimize animal suffering as well as to reduce the number of animals required for experimentation.

### Chronic unpredictable stress model

Chronic unpredictable stress was modified from other models of unpredictable stress^17^,^37^. The animals were divided in two groups: control and stressed. The control group was kept undisturbed in their home cages during the treatment. A variate–stressor paradigm was used for the animals in the stressed group. Individual stressors and length of time applied each day during the week are listed in Supplementary Table S3.

### Procedure

#### Experiment 1: time course of behavior, hippocampal glycogen, and astroglial structural plasticity response to chronic unpredictable stress

Rats were randomly assigned to six experimental groups (*n* = 10/group). Three groups composed the stressed groups, whereas the three other groups composed the control groups. The depression-like behavior of the rats was observed in the sucrose preference test after being stressed for 1, 3, and 5 weeks (Supplementary Fig. S1). Terminal blood samples of the rats (10 controls and 10 stressed) were collected into heparinized tubes after behavioral tests. Plasma was separated by centrifugation at 3000 rpm and stored at −20 °C until the assay of corticosterone concentrations. The rats were then sacrificed for glycogen assay (five controls and five stressed) and morphological analysis (five controls and five stressed).

#### Experiment 2: effects of Gys1 RNAi on astroglial structural plasticity and depression-like behavior

As depicted in Supplementary Fig. S1, animals were divided into two experimental groups as follows. (1) *Control group (n* = 15): the right hippocampus of rats was injected twice with control lentivirus containing negative control (NC) sequence. (2) *RNAi group (n* = 15): the right hippocampus of rats was injected twice with recombinant lentivirus containing shRNA sequence targeting Gys 1. Rats were anesthetized by intraperitoneal injection of 400 mg/kg chloral hydrate and positioned in a stereotaxic apparatus (RLJD68506, RWD Biotechnology Co. Ltd., Shenzhen, China). A 5-μL volume of lentivirus solution (3.02 × 10^9^ integration units per ml) was stereotactically injected into the dentate gyrus region of the right hippocampus at 0.5 μl/min under stereotactic guidance at the following coordinates relative to bregma: AP −3.6 mm, ML 2 mm, and DV 3.6 mm (Supplementary Fig. S2). The needle was held in place for 10 min after injection. The microsyringe was pulled out after the blood had coagulated. The craniotomy was then sealed with bone wax. The scalp was then closed with sutures. The rats were given free access to food and water after they woke up.

Two weeks later, a 5-μL volume of lentivirus solution was stereotactically injected into the CA3 region of the right hippocampus at the following coordinates relative to bregma: AP −3.6 mm, ML 3.6 mm, and DV 3.8 mm (Supplementary Fig. S2). The depression-like behavior of the rats was observed in the sucrose preference test (15 controls and 15 RNAi) three weeks after the second injection. The rats were then sacrificed for silencing efficiency assessment of shRNA against Gys 1 (five controls and five RNAi), glycogen assay (five controls and five RNAi) and morphological analysis (five controls and five RNAi).

#### Experiment 3: effects of chronic stress on hippocampal glycogen metabolism

As depicted in Supplementary Fig. S1, animals were divided into two experimental groups as follows. (1) *Control group (n* = 10): rats were kept undisturbed in their home cages during the treatment. (2) *Chronic stressed group (n* = 10): rats were submitted to an unpredictable stressor daily. The rats were sacrificed to obtain hippocampal tissues for glycogen breakdown and synthesis analysis after being stressed for five weeks. Five animals per group were randomly chosen for biochemical measurement. The remainder of animals was used for adenine nucleotides analysis by high-performance liquid chromatography (HPLC) technique.

### siRNA design, lentivirus production, and lentivirus infection

The NC and recombinant lentivirus was designed and produced by Obio Technology Co., Ltd, Shanghai, China. Briefly, four siRNA sequences targeting Gys 1 (Gene ID NM_001109615) were selected: 5′-CCTTCACGCTCAGAGCAAA (Y2060), 5′-CCTAGACAAGACTTTGTAT (Y2061), 5′-GCTCTACGAATCCCTATTA (Y2062), and 5′-GCGCATCATTCAGCGGAAT (Y2063). The NC sequence was 5′-CCTTCACGCTCAGAGCAAA (Y007). The sense and antisense oligomers were cloned into *Age* I and *EcoR* I restriction sites of lentiviral vector (pLenti-U6-shRNA-CMV-EGFP) to drive the shRNA expression under the U6 promoter. The vector clones were transformed into competent *Escherichia coli.* DH5α was then cultured in LB agar plates to select colonies with inserted shRNA sequences.

Primary cultured rat astrocytes were transfected with the NC or recombinant vectors. Cells were harvested at 48 h after transfection. Using β-actin as an internal standard, a real-time quantitative reverse transcription polymerase chain reaction revealed that the recombinant vector (Y2060) effectively knocked down the mRNA of rat Gys 1 by approximately 80% (Supplementary Fig. S3). This vector was therefore packaged into recombinant lentiviral particles. The lentivirus infection in the hippocampus of rats was observed by EGFP positive expression. The rats were euthanized by an overdose of anesthetic and perfused with 0.9% ice cold saline followed by 4% paraformaldehyde in phosphate buffer after being injected twice with the lentivirus described in Experiment 2. The brain tissues were then removed and serially sectioned (20 μm) on a cryostat. The sections of the injection site were mounted with Fluoromount™ aqueous mounting medium (Sigma) and viewed for green fluorescence under a Zeiss Axio Imager M2 fluorescent microscope.

### Sucrose preference test

During this test, each animal was given a free choice between two bottles for 24 h: one with 1% sucrose solution and the other with tap water. The position of the bottle was switched after 12 h to prevent possible effects of side preference in drinking behavior. No previous food or water deprivation was applied before the test. Sucrose preference is calculated as a percentage of the volume of sucrose intake over the total volume of fluid intake and averaged over the two days of testing.

### Corticosterone assays

Plasma corticosterone was assayed using the AssayMax Corticosterone ELISA kit that employs a quantitative competitive enzyme immunoassay technique (Assaypro, Catalog No. EC3001–1) following the manufacturer’s instructions. The unknown sample concentration was determined from the standard curve. The mean value of the triplicate readings for each sample was calculated.

### Morphological analysis

#### Perfusion and brain tissue preparation

Rats were deeply anesthetized with an overdose of sodium pentobarbital and perfused through the left ventricle with a saline solution followed by 4% paraformaldehyde in phosphate buffer (pH = 7.4). The brains were gently removed, postfixed overnight in the same fixative, and cryoprotected in glycerol/glycol solution. The brain tissue was supported in an optimum cutting tissue medium. The coronal sections were cut at 60 μm thickness along the entire hippocampus according to the rat brain atlas (http://labs.gaidi.ca/rat-brain-atlas/). A total of 54 consecutive sections for each brain tissue were collected free-floating into wells with the saline solution for the following immunohistochemistry and stereological analysis.

#### GFAP immunohistochemistry

Sections were washed thrice with phosphate-buffered saline (PBS) containing 0.1% Triton X-100 (PBST) and blocked with 5% bovine serum albumin and 5% goat serum for 1 h. The sections were incubated with the primary antibody (rabbit polyclonal anti-GFAP, sc-9065, Santa Cruz) overnight at 4 °C. The sections were rinsed several times in PBS the following day, incubated in a polyperoxidase-anti-rabbit IgG (PV9000, ZSGB-BIO, Beijing, China) for 1 h, rinsed again, developed for 5 min in diaminobenzidine, and thoroughly rinsed. The sections were then mounted onto gelatin-coated slides and dried. After that, the sections were dehydrated through stepped alcohol washes, cleared in xylene, and finally coverslipped.

#### Stereological estimates of astrocytic size and protrusion length in the hippocampus

A single examiner who was blind to the group identification performed the data collection. Every sixth section along the hippocampus was selected after randomly selecting a starting point. An average of nine sections per animal was produced for analysis. Selected sections were analyzed. Images were captured with a Zeiss Axio Imager M2 microscope coupled with a Hamamatsu color CCD camera, a computer-controlled X-Y-Z stepping motor system, and a Stereo Investigator software system (MBF Bioscience, Williston, USA). The boundaries of the hippocampal formation were demarcated for the stereological analysis on the GFAP-stained sections using a low power magnification lens.

**Figure 6:**
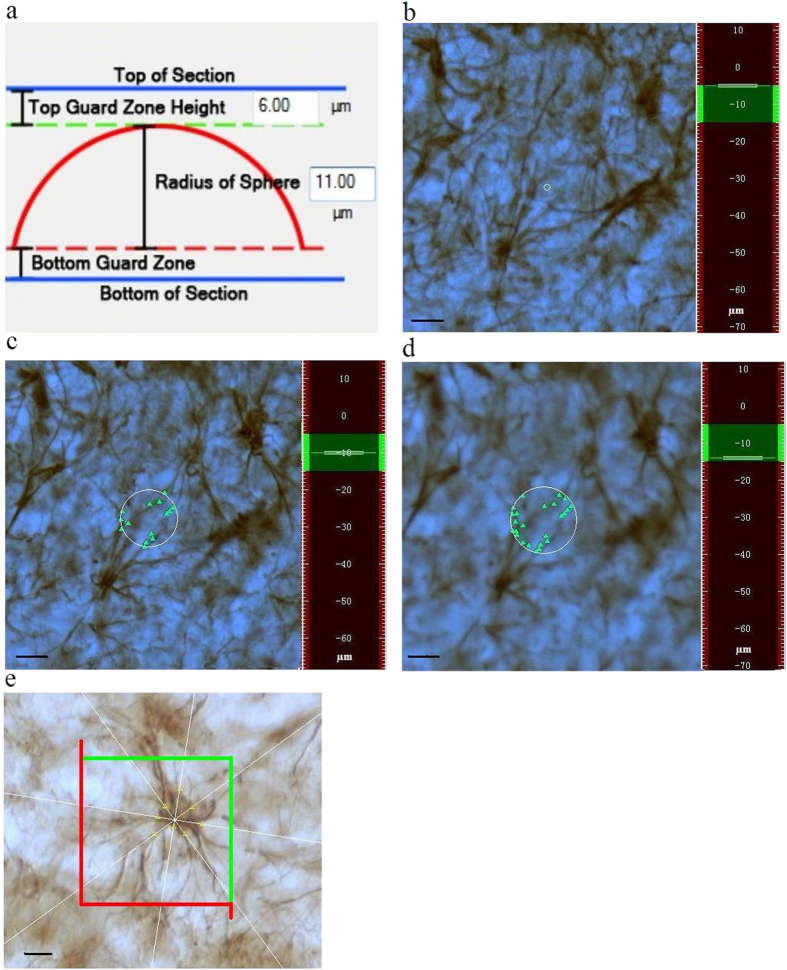
Overview of the application of the hemispherical and nucleator probes. The total length of astrocytic protrusion was estimated using the hemispherical probe (**a**). The virtual hemisphere is visualized as a series of circumferences of changing diameters upon focusing through the tissue (**b**–**d**). The intersections of the circumferences with stained astrocytic protrusion were computed to approximate the total length of astrocytic protrusion. The estimate of cell size was achieved using the vertical nucleator probe, which randomly generated measuring rays from the center to the edge of the cells (**e**). The scale bar is 10 μm.

A hemispherical probe was used to estimate the total length of astrocytic protrusion (Fig. 6a). Pilot studies were used to calculate the average thickness of the sections post-processing (23 μm) to set appropriate virtual hemispheres (radius of 11 μm) and guard zones (6 μm). An XY grid of 484 μm × 360 μm was set, and 60 XY grids were defined within the maximum sampling area of the hippocampus. The software automatically generates virtual hemispheres, which are visualized as a series of circles of changing circumferences upon focusing through the tissue (Fig. 6b–d). The number of intersections of stained astrocytic protrusion with each circumference was counted following the stereological principles of systematic random sampling. The total length of astrocytic protrusion was calculated according to the counted intersections and defined sampling parameters. The precision of the protrusion length estimate was expressed as the coefficient of error.

The nucleator probe was used to estimate the size of each astrocyte whose cell body fulfilled the 3D counting rules. Briefly, a set of eight rays were extended from a point within the cell and radiated with a random orientation toward the edge of the profile at the largest cross-sectional profile of the cell. The eight intersections with the cell boundary were marked. The somal volume was automatically calculated by the software (Fig. 6e). The somal volume was averaged over at least 270 astrocytes randomly selected within nine sections under the guidance of a counting frame (50 μm × 50 μm).

### Biochemical measurement

#### Hippocampal tissue preparation

As for glycogen and adenine nucleotides analysis, rats were sacrificed using a high energy (10 kW) focused microwave irradiation (Gerling Applied Engineering Inc.) for 1.2 sec, which inactivates most brain enzymes before extraction or digestion^38^. Regarding other biochemical measurement, rats were anesthetized using sodium pentobarbital and flashfrozen through rapid immersion in liquid nitrogen. The skull was then opened, and the brain tissues were removed rapidly from the skulls. The hippocampus was separated from the cortex covering the skull along the surface toward the ventral part of the hippocampus. The frozen hippocampal tissues were pulverized under liquid nitrogen using a mortar and a pestle for the following biochemical analysis.

#### Glycogen assay

Tissue glycogen was assayed using the glycogen assay kit (Abnova, Catalog No. KA0861). In the assay, glucoamylase hydrolyzes the glycogen to glucose, which was then specifically oxidized to produce a product that reacts with the OxiRid probe to generate color (optical density of 570 nm). The powdered hippocampal tissues were homogenized in boiling water, and centrifuged at 18,000 rpm for 10 min following the assay protocol. The supernatant was then ready for assay. The amount of glycogen in unknown samples was calculated using the standard curve of glycogen (MW ~10^6^–10^7^ Da) and was expressed as μg glycogen per mg hippocampal tissue. The standard curve was *y* = 0.3683 *x* + 0.0003 [*y* = A_570_; *x* = glycogen amount (μg)].

#### Enzyme activity assay

The powdered hippocampal tissues were homogenized in ice-cold lysis buffer and centrifuged at 8000 rpm for 10 min. The supernatants were then used for activity assay of Gys 1 and Gyp^39^. The detailed methods and principles are described in the Supplementary Methods. Gys 1 activity was expressed as nmol uridine diphosphate (UDP)/mg hippocampal tissue/min. Gyp activity was expressed as ΔA_340_/mg hippocampal tissue/min.

#### Western Blot analysis

The powdered hippocampal tissues were homogenized in ice-cold lysis buffer. Aliquots of the clarified homogenized liquid containing 75 μg of protein were denatured and analyzed by 12% sodium dodecyl sulfate-polyacrylamide gel electrophoresis and transferred to polyvinylidene fluoride membranes (Bio-Rad). The primary antibodies included rabbit monoclonal anti-Gys 1 (1:2000, Abcam, ab40810), mouse monoclonal anti-Gyp (1:2000, Abcam, ab88078), and mouse monoclonal anti-β-actin (1:2000, Sigma, A1978). The secondary antibodies were horseradish peroxidase (HRP)-conjugated goat anti-mouse IgG (1:4000, GeneScript) or goat anti-rabbit IgG (1:4000, GeneScript). Immunoblotting was detected by enhanced chemiluminescence (Amersham) and analyzed using an FR-200A Electrophoresis Image Analysis System (Furi, Shanghai, China). The values of Gys 1 and Gyp levels were normalized against the amount of β-actin obtained from the same sample.

#### Real-time polymerase chain reaction (PCR) analysis

Total RNA from powdered hippocampal tissues was extracted using Trizol reagent (Invitrogen). cDNA was synthesized with 2 μg of total RNA using the RevertAid™ Transcript First-Strand cDNA Synthesis Kit (Fermentas, K1622). Quantitative real-time PCR was performed using the SYBR Green Master Mix (Fermentas, K0222) in the StepOne™ Real-Time PCR System (ABI, USA). The PCR primers and thermal cycling conditions are described in Supplementary Table S4. The mRNA relative expression of Gys 1 or Gyp was calculated using the 2^−ΔΔCt^ method [ΔCt = Ct(the target gene) − Ct(GAPDH)].

#### NanoPro immunoassay

The NanoPro immunoassay method involves separation of tissue lysates in the capillary isoelectric focusing step, followed by analysis of protein phosphorylation (NanoPro 1000 system, Protein Simple, Santa Clara, USA). Powdered hippocampal tissues (100 mg) were homogenized in 500 μl Bicine/CHAPS lysis buffer (Protein Simple, 040–764) plus 10 μl dimethyl sulfoxide inhibitor mix (Protein Simple, 040–510) and 20 μl aqueous inhibitor mix (Protein Simple, 040–482). The tissue lysates were cleared by centrifugation at 18,000 rpm for 1 h at 4 °C. When indicated, tissue lysate was incubated with calf intestinal phosphatase (New England Biolabs, M0290S) at 37 °C for 1 h. The supernatants were diluted to 1000 μg/μl by sample diluents (Protein Simple, 040–649) in the presence of a dimethyl sulfoxide inhibitor mix (Protein Simple, 040–510). Diluted supernatants were combined with Premix G2 (Protein Simple, 040–968) and PI standard ladder 1 (Protein Simple, 040–968). The charge-based separation was performed in a capillary at 45,000 W for 40 min. Immobilization was conducted with 100 s of irradiation with UV light. The sample was incubated with the primary antibody described by the Western blot analysis for 120 min after separation and immobilization. It was then incubated with biotin-conjugated goat anti-mouse or goat anti-rabbit IgG (Protein Simple, 041–127 or 041–126) for 1 h followed by 10 min of incubation with streptavidin-conjugated HRP. Chemiluminescence signals for the target proteins were detected by adding luminol (Protein Simple, 040–652) and peroxide XDR detection reagents (Protein Simple, 041–084). They were then analyzed with the Compass software.

#### Determination of ATP, ADP, and AMP in the hippocampus

The protocol to determine ATP, ADP, and AMP was modified from Di Pierro *et al*.^40^. The powdered hippocampal tissues were homogenized with 0.5 ml of 0.2 M perchloric acid. The homogenate was centrifuged at 12,000 rpm for 15 min. The resultant supernatant was neutralized with a 4 M NaOH solution. Up to 20 μl of the supernatant was used for the ion-pairing HPLC-UV analysis. The HPLC system, chromatographic separation conditions, and representative chromatograms are described in Supplementary Fig. S4. The concentrations of ATP, ADP, and AMP in the supernatant were calculated from standard curves. The ATP, ADP, and AMP levels in the hippocampus were expressed as nmol/mg tissue.

### Statistical analysis

Data were expressed as mean ± S.E.M. for the indicated number of experiments and analyzed using the Statistical Package for Social Sciences computer program version 10.1. As for Experiments 1 and 2, statistical significance was determined by a two-way ANOVA, followed by a Student–Newman–Keuls *post hoc* analysis for further examination of group differences. A two-tailed Student’s *t*-test was used for Experiment 3. The significance level was set at *P* ≤ 0.05 for all statistical comparisons.

## Additional Information

**How to cite this article:** Zhao, Y. *et al*. Decreased Glycogen Content Might Contribute to Chronic Stress-Induced Atrophy of Hippocampal Astrocyte volume and Depression-like Behavior in Rats. *Sci. Rep.* **7**, 43192; doi: 10.1038/srep43192 (2017).

**Publisher's note:** Springer Nature remains neutral with regard to jurisdictional claims in published maps and institutional affiliations.

## Supplementary Material

Supplementary Data
