# Supplementary material for: Decreased Glycogen Content Might Contribute to Chronic Stress-Induced Atrophy of Hippocampal Astrocyte volume and Depression-like Behavior in Rats
Source: Sci Rep. 2017 Feb 24;7:43192. doi: 10.1038/srep43192 (PMC5324119; doi:10.1038/srep43192)
Supplement: Supplementary Data [file srep43192-s1.pdf]

Decreased Glycogen Content Might Contribute to Chronic Stress-Induced Atrophy of Hippocampal Astrocyte volume and Depression-like Behavior in Rats

Yunan Zhao*, Qiang Zhang, Xiao Shao, Liufeng Ouyang, Xin Wang, Kexuan Zhu, and Lin Chen

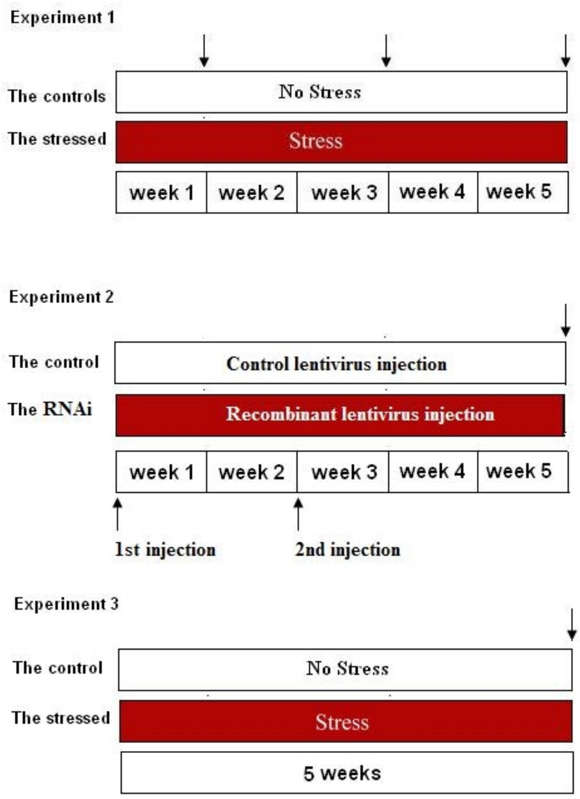

Supplementary Fig. S1. Experimental groups and design. For details, see the Materials and methods section. Symbol "↓" means that the rats were decapitated to obtain venous blood and/or brain tissues after behavioral tests.

* Corresponding author: Tel/Fax: +86-24-85811922. E-mail: zhaoyunan-js@163.com (Y. Zhao)

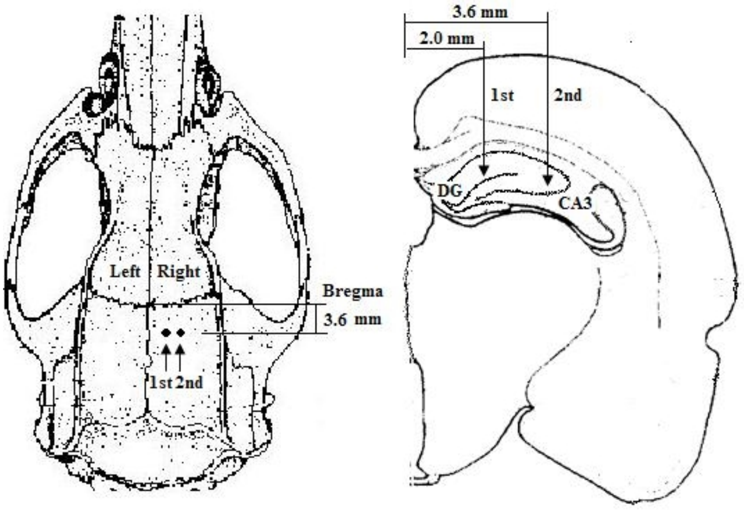

Supplementary Fig. S2. Stereotactic injection diagram of right hippocampus.

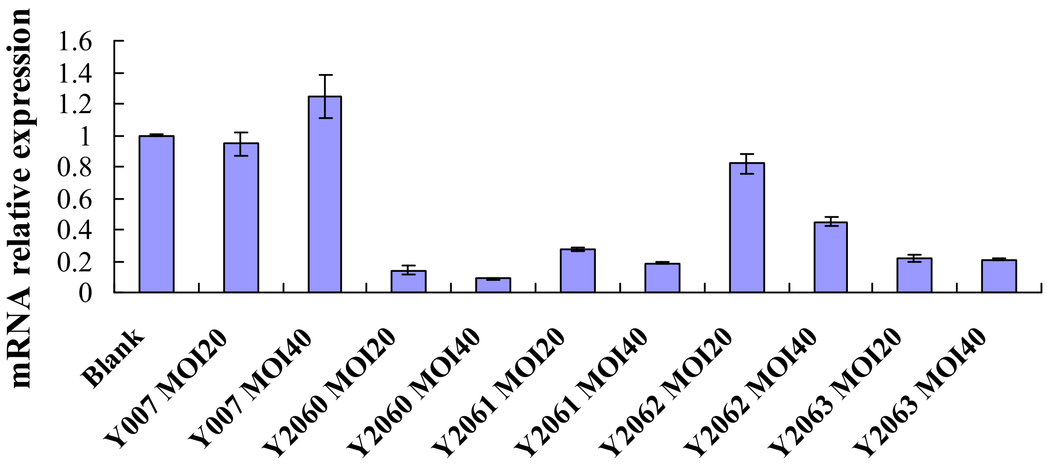

Supplementary Fig. S3. Effects of the control (Y007) and recombinant (Y2060~Y2063) vectors on Gys 1 mRNA expression in astrocytes cultured *in vitro*.

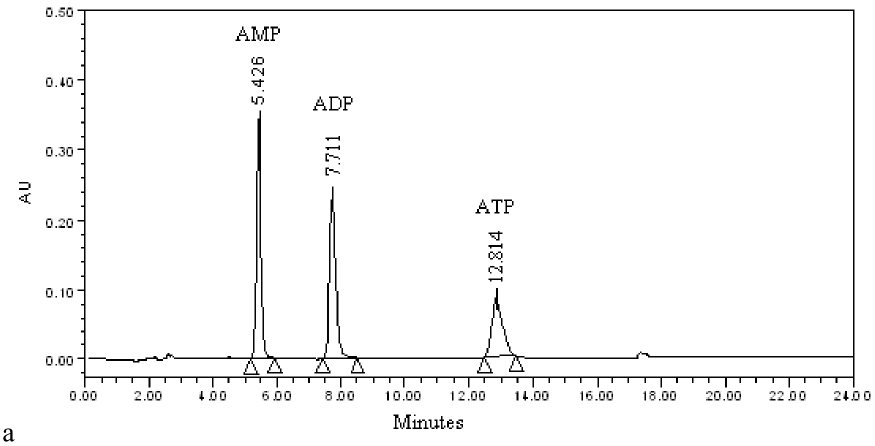

a

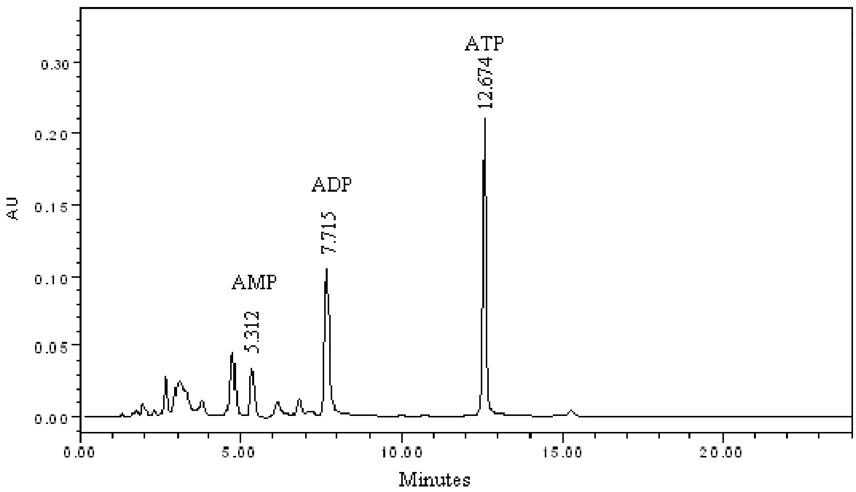

b

Supplementary Fig. S4. Representative HPLC-UV chromatograms of AMP, ADP, and ATP reference standards (a) and hippocampal tissue samples (b). Samples were analyzed using a Dionex Ultimate 3000 UHPLC+ Focused system equipped with a 3000RS pump, a 3000RS autosampler, a 3000RS column compartment, and a 3000RS diode array detection (DAD) system. A Supelcosil LC-18 column (150 mm × 4.6 mm i.d.; particle size, 5 μm) was used with a column oven heated to 25 °C. The mobile phase consisted of 0.1 M phosphate buffer (containing 1 mM tetrabutylammonium hydroxide solution)-acetonitrile (97.5:2.5, v/v; pH 6.0). The flow rate was 1 ml/min and the detector was set at 254 nm. The standard curves for ATP, ADP and AMP were y=34,199x, y=31,715x and y=32,198x, respectively [y=peak area; x=concentration (μM)].

Supplementary Table S1. Detailed individual data of astrocyte somal volume and protrusion length.

Week	Animal number	Treatment	Somal volume mean ± S.E.M. (μm^3)	Protrusion length $\times 10^8$ μm	CE[a]
1	c1	control	104 ± 12	2.900	0.03
	c2	control	113 ± 21	2.341	0.04
	c3	control	97 ± 17	2.416	0.03
	c4	control	94 ± 19	2.939	0.04
	c5	control	89 ± 23	2.255	0.04
	s1	stress	101 ± 18	2.358	0.03
	s2	stress	94 ± 16	2.991	0.03
	s3	stress	107 ± 22	2.794	0.03
	s4	stress	95 ± 17	2.685	0.04
	s5	stress	90 ± 21	2.543	0.04
3	c1	control	91 ± 11	2.807	0.04
	c2	control	95± 11	2.431	0.04
	c3	control	98 ± 12	2.519	0.03
	c4	control	106 ± 14	2.945	0.04
	c5	control	108± 21	2.354	0.03
	s1	stress	71 ± 16	2.251	0.03
	s2	stress	89 ± 16	2.697	0.04
	s3	stress	95 ± 18	2.094	0.03
	s4	stress	95 ± 22	2.355	0.03
	s5	stress	75 ± 17	2.034	0.04
5	c1	control	117 ± 25	2.919	0.03
	c2	control	101 ± 18	2.514	0.03
	c3	control	88 ± 16	2.218	0.03
	c4	control	91 ± 19	2.829	0.04
	c5	control	89 ± 23	2.355	0.04
	s1	stress	66 ± 22	2.151	0.04
	s2	stress	78 ± 11	2.476	0.03
	s3	stress	84 ± 25	1.804	0.04
	s4	stress	88 ± 23	2.281	0.04
	s5	stress	71 ± 21	1.878	0.03

[a] The precision of the protrusion length estimate was expressed as the coefficient of error (CE).

Supplementary Table S2. Detailed individual data of astrocyte somal volume and protrusion length.

Group	Animal number (hippocampus)	Treatment	Somal volume	Protrusion length	
			mean ± S.E.M. (μm^3)	$\times 10^8$ μm	CE[a]
Control	C1(left)	Untreated	100 ± 11	2.100	0.04
	C2(left)	Untreated	108 ± 18	2.731	0.03
	C3(left)	Untreated	94 ± 17	2.926	0.03
	C4(left)	Untreated	90 ± 14	2.487	0.03
	C5(left)	Untreated	85 ± 21	2.851	0.04
	C1(right)	shRNA (NC)	127 ± 11	2.957	0.04
	C2(right)	shRNA (NC)	98± 19	2.911	0.04
	C3(right)	shRNA (NC)	120 ± 21	2.511	0.03
	C4(right)	shRNA (NC)	109 ± 16	2.187	0.04
	C5(right)	shRNA (NC)	104 ± 20	2.241	0.03
RNAi	R1(left)	Untreated	94 ± 10	2.823	0.03
	R2(left)	Untreated	100± 12	2.136	0.03
	R3(left)	Untreated	96 ± 17	2.712	0.04
	R4(left)	Untreated	107 ± 15	3.012	0.04
	R5(left)	Untreated	92± 21	2.154	0.04
	R1(right)	shRNA (Gys1)	70 ± 21	2.117	0.03
	R2(right)	shRNA (Gys1)	74 ± 19	2.591	0.03
	R3(right)	shRNA (Gys1)	87 ± 23	2.521	0.03
	R4(right)	shRNA (Gys1)	94 ± 14	2.951	0.04
	R5(right)	shRNA (Gys1)	86 ± 21	1.908	0.03

[a] The precision of the protrusion length estimate was expressed as the coefficient of error (CE).

Supplementary Table S3. One week representative schedule[a] of stressor agents used during the treatment.

Day of treatment	Stressor	Duration
1	Shaking	15 min
2	No stressor applied	–
3	Flashing light[b]	3 h
4	Swimming in cold water	15 min
5	Restraint[c]	6 h
6	Footshock[d]	20 min
7	Wet sawdust	24 h

[a] Schedule for each week was randomly generated by the use of above seven stressor agents.

Stress application started at different times everyday, in order to minimize its predictability.

[b] Exposure to flashing light was made by placing the animal in a 25 cm-high, 45 cm × 30 cm open field made of brown plywood with a frontal glass wall. A 40 W lamp, flashing at a frequency of 60 flashes/min, was used.

[c] Restraint was carried out by placing the animal in a 5 cm × 15 cm plastic tube and adjusting it with plaster tape on the outside, so that the animal was unable to move. There was a 1 cm hole at the far end for breathing.

[d] Footshocks were administered through the grid floor of shock boxes (15 cm × 18 cm, NatureGene Corp., Beijing, China). A random shock generator was used to deliver about 20 shocks of 0.2 mA, 1-s duration, within a 20 min period.

Supplementary Table S4. Primers for PCR and thermal cycling conditions[a].

	Forward	Reverse
Gys 1	5′-CGCACAGAGCGATTGTCAG-3′	5′-GGTACCCCTGGGTCGCAT-3′
Gyp	5′-GGACCAGCGGGCATTTGTTTTC-3′	5′-ACTAGGAGCACTTCTAGCACATTCC-3′
GAPDH	5′-TGCACCACCAACTGCTTAGC-3′	5′-GGCATGGACTGTGGTCATGA-3′

[a] Initial denaturation at 95 °C for 5 min; 40 cycles of 95 °C for 10 s, 60 °C for 30 s; cooled to 4°C.

Supplementary Methods

1. Gys 1 activity assay

1.1 Principles

Reaction1: $\text{glucose}_n + \text{UDPG} \xrightarrow{GS} \text{UDP} + \text{glucose}_{n+1}$

Reaction2: $\text{PEP} + \text{UDP} \xrightarrow{PK} \text{pyruvate} + \text{UTP}$

Reaction3: $\text{pyruvate} + \text{NADH} \xrightarrow{LDH} \text{lactate} + \text{NAD}^+$

GS: Glycogen synthase 1; PEP: phospho(enol) pyruvate
PK: pyruvate kinase; LDH: lactate dehydrogenase

1.2 Methods

Gys 1 activity was detected in 96-well plates, and 20 µl of supernatant or UDP standard solution was added to 50 µl reaction solution A (50 mM Tris, 1 mM EDTA, 1 mM NaF, 0.5 mM DTT, 0.02 mM BSA, 2mM UDPG, pH ≈ 7.4), for an incubation of 60 min at 38℃, followed by adding 10 µl of 0.5 M NaOH solution to stop the reaction. 1 ml of reaction solution B (50 mM Tris, 50 mM KCl, 4 mM $MgCl_2$, 100 µM phospho (enol) pyruvate, 8 µM NADH, 3 U/ml pyruvate kinase, 0.25 U/ml lactate dehydrogenase, 65 U/ml catalase, 0.02% BSA, pH ≈ 6.7) was then added to each well and incubated for 20 min at room temperature. The plates were laid in a fluorescence plate reader (SpectraMax M5, Molecular Devices, USA) and NAD^+ formation was measured at 340 nm excitation and 460 nm emission. The generated UDP in reaction solution A was calculated using the standard curve of UDP [$y = -0.03355\,x + 2.3673$, $x = A_{460}$, y = UDP concentraion (mM)]. Gys 1 activity was expressed as nmol UDP/mg hippocampal tissue/min.

2. Gyp activity assay

2.1 Principles

$$\text{Reaction1: glucose}_n\text{+Pi} \xrightarrow{\textit{phosphorylase a}} \text{glucose-1-P+glucose}_{n-1}$$

$$\text{Reaction2: glucose-1-P} \xrightarrow{\textit{PGluM}} \text{glucose-6-P}$$

$$\text{Reaction3: glucose-6-P+NADP}^+ \xrightarrow{\textit{G6PDH}} \text{gluconolactone-6-P+NADPH+H}^+$$

PGluM: phosphoglucomutase; G6PDH: glucose-6-phosphate dehydrogenase

2.2 Methods

Gyp activity was also detected in 96-well plates, and 20 μl of supernatant was added into 180 μl reaction solution (18 mM KH_2PO_4、27 mM Na_2HPO_4、1.4 mM $MgCl_2$、0.8 mM EDTA、0.4 mM $NADP^+$、4 mM glucose-1,6-bisphosphate, 1.7 U/ml glucose-6-phosphate dehydrogenase, 1 U/ml phosphoglucomutase, pH ≈ 6.8). The reaction was activated with 2 mg/ml glycogen, and incubated at 37℃. The change in the absorbance at 340 nm was monitored every 2 minutes. Gyp activity was expressed as ⊿A_{340}/mg hippocampal tissue/min.
